# Cases Extracted from the Note-Book of Henry Davies, M.D.

**Published:** 1835-07-01

**Authors:** 


### ORIGINAL COMMUNICATIONS.

---

*Cases extracted from the Note-book of* Henry Davies, M.D. Physician to the British Lying-in Hospital, &c.

### CASES OF ERYSIPELAS TREATED WITH THE NITRATE OF SILVER.

### 1. *Erysipelas and Purpura.*

Mrs. M., ætat. twenty-seven, residing in Gray's Inn lane, ten weeks after her confinement with her fifth child (which she suckled), finding that she recovered but slowly, went out of town to recruit her health. While on the water in an open boat, she was very much alarmed at the approach of a steam-boat, and on her return to land as quickly as possible, she had a violent rigor, accompanied with syncope and succeeded by fever; the extremities became swollen, and her throat sore. She was bled twice, had leeches to the throat, and took aperients and saline medicine.

Sunday, June 15, 1834. Some discoloration of the integuments of the fore-arm.

Monday, June 16. Saw her for the first time, in consultation with Mr. M'Donald. The left arm and hand appeared as if severely contused and ecchymosed; they were swollen and tense, from the tips of the fingers to a little above the elbow, where the tumour was bounded by an irregular but defined margin; there were some vesications about the fingers. The right-hand and arm were somewhat puffy, without ecchymosis, but there were three or four irregular, dark-coloured blotches, resembling purpura hemorrhagica, together with a similar patch on the forehead, one surrounding the left eye (with suffusion also on the conjunctiva), and a third very large patch in great part surrounding the mouth. These patches were unaccompanied by any tumefaction whatever, appearing simply as effused blood, and their appearance was unaltered by pressure. The pulse was feeble and frequent, 120—130; heat of the skin moderate; extreme languor and prostration of strength; voice feeble; deglutition difficult; fauces and back of the throat purple and swollen; tongue pale and coated. The left arm resembled somewhat Bateman's plate of Erythema marginatum, except that it was darker; the patches of the other parts resembled that of purpura. The patient was ordered to take a draught of salts and senna immediately, and an ammoniated saline draught, with gr. iii. of excess of carbonate of ammonia, every four hours; and the following refrigerant lotion to be assiduously applied to the arm by means of linen. ℞. Liq. Ammon. Acet. ℥iv.; Sp. Vin. Rect. ℥ij.; Aq. Rosæ ℥ij.; M. ft. Lotio. Weak beef-tea;—the infant to be applied to the breasts only sufficiently often to relieve their tension.

Monday evening. Has had two alvine evacuations, in which there is nothing unnatural; is extremely weak. The medicine to be continued, with the addition of twenty minims of Liq. Opii sed. at bed-time.

Tuesday, 17th. Pulse less frequent; voice somewhat better; local symptoms stationary. Ineffectual endeavours had been made to remove her ring on the Sunday, and it now caused so much pain that it was necessary to file it off.

Wednesday. Mouth and throat very sore; pulse 120, feeble; feet and legs cold; some wine and water was prescribed with a little brandy, in arrowroot or sago; a foot-warmer was ordered to be applied to the feet, and they were to be placed in a mustard bath at night. Rep. Haust. Ammon. et Haust. Anodyn. et adde Ext. Papaveris ʒij. lotioni.

Thursday. Passed a good night; copious moisture on the skin; speaks better; tongue clammy and pale; mouth very sore, cannot open it sufficiently to allow the throat to be accurately examined; a good deal of stiffness round the right shoulder, which is very tender to the touch; ecchymosis extending upwards; the hand is less swollen; the fingers can be moved; discoloration more mottled in appearance; the patches on the face and those on the right arm are less florid, resembling the colour of the outer side of oyster-shells; the tips of the fingers ecchymosed; skin shrivelled, as if the hand had been soaked in water, and blood is effused under the surface. A pencil of Argentum Nitratum was moistened, and well rubbed round to the extent of an inch beyond the margin of the ecchymosis of the left arm; the senna draught has operated; dejections natural. Add gr. ii. of Amm. carb. and ℥i. Infus. Cascar. to each draught. Repeat the anodyne, and continue lotion with Ext. Papaveris.

Friday, 20th. Is generally improved; a deep ulcer on the left tonsil, and sloughing of the uvula. Cont. Med.

Sunday, 22d. The ecchymosis had not extended beyond the cauterized boundary, from which there was a moist exudation; the integuments of the arm were softer and compressible, the colour brown and mottled; the shrivelled appearance of the hands had disappeared, and spots on the other parts were merely of a dusky or dirty colour; throat and mouth still sore, but improving. The patient cannot swallow anything but liquids; tongue cleaner; heat natural; pulse 96—100, very feeble. She was ordered to continue the medicines; to take coffee and tea with cream, and a little good ale, which she asked for.

June 25th. Countenance cheerful; complexion clear, and also right arm, except a dusky appearance at the bend of the elbow; the skin of the left arm desquamating in brown scales; pulse eighty; tongue clean; appetite moderate.

June 30. Improving; light tonics with ammonia; an occasional purgative; a stimulant embrocation to the arm, and generous diet. In a few days she was well.

In this case may be remarked, 1st. The extremely slow progress towards recovery which occasionally characterizes the puerperal state, without any manifest cause, and which is commonly symptomatic of disease in some of the deep-seated tissues of the pelvic viscera. 2dly. The supervention of the erysipelatous and purpuragic form of disease, immediately subsequent to the violent mental impression characteristic of the broken down state of the constitution, and loss of tone of the capillaries. And lastly, The utility of the nitrate of silver in arresting the progress of the erysipelas upwards. Its power is more fully exemplified in the subjoined cases.

2. *Erysipelas supervening on Pneumonia.*

I was requested to meet Mr. Bryant, of the Edgeware-road, April 4, 1830, to see Mrs. S., aged forty-eight, very stout, and generally healthy. She had had a severe attack of pneumonia, for which she had been largely bled, leeched, &c. &c. There was still some dyspnœa; a rapid vibratory pulse; foul tongue; extreme prostration of strength; and an erysipelatous blush on the skin of the face. She was purged; took calomel with opium, and a saline mixture with gr. ii. excess of ammonia every four hours.

April 6th. Her breathing was relieved, and her tongue cleaner; but the erysipelas now extended over the nose and eyelids; extremities cold; nausea and vomiting violent. This was eventually relieved by the cretaceous mixture, combined with Tr. Opii.

April 8th. Has kept down some veal-tea; pulse less vibratory, feeble; tongue clean, florid; the whole face swollen, with part of the scalp and throat. The eyes were closed, and she was comatose and delirious. She was ordered to continue the ammoniated draught, and to have mustard pediluvia; with whey, and beef-tea for diet. On the proposal of Mr. Bryant the whole of the erysipelatous surface was rubbed over with lunar caustic, the application being extended beyond the margin of the diseased surface. The progress of the erysipelas the next day seemed checked; but from having rubbed her face with her hands, and in her comatose delirious condition, her appearance was like anything but a woman in a favourable state. By continuing to use the carbonate of ammonia, with a mild nutritious diet, and preserving regularity of bowels, she gradually amended, and, at the end of a fortnight from the application of the caustic, she had quite recovered.

### 3. *Erysipelas from a Contusion.*

Dec. 1830. I was requested to meet Mr. Holton, of Lisson-grove, North, to see Mr. ——, who, in getting from a coach, had received a slight contusion of the integuments of the right temple. This was considered, at the time, of little importance, but a few days afterwards he had an attack of fever, accompanied by sore-throat, and succeeded by erysipelas of the face and part of the scalp, extending to the ears and neck. The whole was much swollen; he was delirious and comatose, with cold extremities, and extreme prostration of strength. In addition to the medicinal means employed, the whole erysipelatous surface was rubbed with Argentum Nitratum. From the projection of the beard and whiskers, the caustic had not got, in every part, so immediately into contact with the surface as was desirable; it was therefore reapplied on the following day, in the form of solution. The progress of the erysipelas was arrested; the power of deglutition was also improved; and from this time, assisted by appropriate medicine and diet, he gradually recovered.

---

